# PRAME promotes epithelial-to-mesenchymal transition in triple negative breast cancer

**DOI:** 10.1186/s12967-018-1757-3

**Published:** 2019-01-03

**Authors:** Ghaneya Al-Khadairi, Adviti Naik, Remy Thomas, Boshra Al-Sulaiti, Shaheen Rizly, Julie Decock

**Affiliations:** 1 College of Health and Life Sciences (CHLS), Hamad Bin Khalifa University (HBKU), Doha, Qatar; 2 Cancer Research Center, Qatar Biomedical Research Institute (QBRI), Qatar Foundation, Doha, Qatar

**Keywords:** PRAME, PReferentially Antigen expressed in Melanoma, Triple negative breast cancer, Migration, Invasion, Epithelial-to-mesenchymal transition

## Abstract

**Background:**

The triple negative breast cancer (TNBC) paradox marks a major challenge in the treatment-decision making process. TNBC patients generally respond better to neoadjuvant chemotherapy compared to other breast cancer patients; however, they have a substantial higher risk of disease recurrence. We evaluated the expression of the tumor-associated antigen PReferentially Antigen expressed in MElanoma (PRAME) as a prognostic biomarker in breast cancer and explored its role in cell migration and invasion, key hallmarks of progressive and metastatic disease.

**Methods:**

TCGA and GTeX datasets were interrogated to assess the expression of PRAME in relation to overall and disease-free survival. The role of PRAME in cell migration and invasion was investigated using gain- and loss-of-function TNBC cell line models.

**Results:**

We show that PRAME promotes migration and invasion of TNBC cells through changes in expression of E-cadherin, N-cadherin, vimentin and ZEB1, core markers of an epithelial-to-mesenchymal transition. Mechanistic analysis of PRAME-overexpressing cells showed an upregulation of 11 genes (*SNAI1*, *TCF4*, *TWIST1*, *FOXC2*, *IL1RN*, *MMP2*, *SOX10*, *WNT11*, *MMP3*, *PDGFRB*, and *JAG1*) and downregulation of 2 genes (*BMP7* and *TSPAN13*). Gene ontology analyses revealed enrichment of genes that are dysregulated in ovarian and esophageal cancer and are involved in transcription and apoptosis. In line with this, interrogation of TCGA and GTEx data demonstrated an increased PRAME expression in ovarian and esophageal tumor tissues in addition to breast tumors where it is associated with worse survival.

**Conclusions:**

Our findings indicate that PRAME plays a tumor-promoting role in triple negative breast cancer by increasing cancer cell motility through EMT-gene reprogramming. Therefore, PRAME could serve as a prognostic biomarker and/or therapeutic target in TNBC.

## Background

Breast cancer remains a major health burden worldwide, despite many improvements in diagnostics and development of a wide range of novel treatment options. It remains the most commonly diagnosed cancer among women, affecting one out of four, and is the second leading cause of cancer-related deaths after lung cancer [1]. Breast cancer is a heterogeneous disease and requires tailored treatment based on distinct tumor features. Based on the expression of 3 biomarkers (estrogen receptor ER, progesterone receptor PR and human epidermal growth factor receptor Her2), we can classify breast cancer into 4 major intrinsic molecular subtypes; luminal A (ER + or PR + , Her2 −), luminal B (ER + or PR + , Her2 +), Her2 + (ER −, PR −, Her2 +) and triple negative (ER −, PR −, Her2 −) breast cancer [2, 3]. Each of these subtypes is characterized by a distinct gene expression profile, and is associated with a different prognosis and metastasis profile. Triple negative breast cancer (TNBC) is associated with the worst clinical outcome with patients facing an excess mortality accounting for one-third of all breast-cancer related deaths [4]. The poor prognosis of TNBC results from its inherent aggressive nature and from the lack of specific therapeutic options for these patients. Systemic therapy is limited to standard anthracycline and taxane-based chemotherapy since the absence of ER, PR and Her2 expression precludes targeted therapy. Despite a complete pathological response in 30% of patients, the majority will relapse and develop brain and lung metastases within 3–5 years after diagnosis [5]. These poor prospects justify the need for new targeted treatments for TNBC and validate the ongoing efforts to identify novel biomarkers for residual disease and metastasis. Several promising therapeutic agents are currently in clinical trial, including Poly (ADP-ribose) Polymerase (PARP) inhibitors, Src inhibitors, and anti-angiogenic and anti-Epidermal Growth Factor Receptor (EGFR) agents [6]. However, none of these regimens are effective in treating all cancer cases and further studies are undertaken to identify subgroups of patients that may benefit more from targeted treatments. Although TNBC is clinically treated as 1 subtype of breast cancer, molecularly it can be further subdivided into 6 groups; a basal-like 1, basal-like 2, immunomodulatory, mesenchymal, mesenchymal-stem like and a luminal androgen receptor group [7]. It has been reported that the rate of pathological complete response after neo-adjuvant chemotherapy differs greatly between TNBC subgroups, suggesting that the residual disease burden may vary considerably between subtypes thereby conferring different risks of relapse and metastasis [8]. Hence, the search for biomarkers to predict the presence of minimal residual disease and the risk of metastasis remain the focus of many studies on triple negative breast cancer.

PReferentially Antigen expressed in MElanoma (PRAME), otherwise known as cancer testis antigen 130 (CT130), MAPE (melanoma antigen preferentially expressed in tumors) and OIP4 (OPA-interacting protein 4) is a member of the cancer testis antigen (CTA) family. PRAME expression in normal somatic tissues is epigenetically restricted to adult germ cells with low expression in the testis, epididymis, endometrium, ovaries and adrenal glands [9, 10]. Similar to the CTA member NY-ESO-1, PRAME was identified as an immunogenic tumor-associated antigen in melanoma, and since its discovery its expression has been demonstrated in a variety of solid and hematological malignancies including triple negative breast cancer [11–20]. Several studies showed that upregulation of PRAME expression in various types of malignancies appears to be linked to promoter DNA hypomethylation, in which treatment with the DNA methyltransferase inhibitors including 5-aza-2′-deoxycytidine (5-azaC) induces PRAME expression [9, 21–23]. A recent study showed that PRAME expression can be induced by the transcription factor myeloid zinc finger 1 (MZF1) in cooperation with DNA hypomethylation, which can be further enhanced using 5-azaC [24]. Aberrant expression of PRAME has been associated with poor prognosis and increased risk of metastasis in many solid tumors, whereas it has been found to predict a more favorable outcome in acute myeloid and lymphoblastic leukemia [25–32]. Although PRAME expression has been linked with poor survival and a higher risk of metastasis in solid tumors, its biological role in cancer is not well understood. Experimental evidence in melanoma demonstrates that PRAME is a dominant repressor of the retinoic acid signaling pathway, thereby inhibiting retinoic acid-induced differentiation, cell cycle arrest and apoptosis [33]. Additional reports in a wide range of cancers suggest that PRAME induces cell proliferation, inhibits apoptosis and reduces cytotoxic drug sensitivity [34–37]. To date, very little data is available on the role of PRAME in regulating metastasis. Contrary to the clinical association of PRAME expression with increased risk of metastasis, the sparse experimental evidence suggests that PRAME inhibits metastasis, albeit the underlying mechanisms remain elusive [38, 39].

Therefore, the present study aimed to provide more compelling data on the role of PRAME in metastasis. More specifically, we explored the molecular function of PRAME in cell migration and invasion of triple negative breast cancer cells. This is the first in vitro study to investigate the role of PRAME in metastasis of triple negative breast cancer by manipulating its expression using 2 different approaches, either by silencing or overexpressing PRAME. We found that PRAME significantly increases both the migratory and invasive potential of triple negative breast cancer cells. In addition, we show here that PRAME is involved in the regulation of the epithelial-to-mesenchymal transition (EMT), as demonstrated by changes in several EMT-associated genes. PRAME overexpression increased the protein expression of the mesenchymal marker vimentin, and induced a redistribution of the epithelial cell–cell adhesion protein E-cadherin from the cell surface to punctate intracellular structures. In addition, PRAME induced the expression of the key EMT-driver ZEB1 and of several EMT-associated genes that are involved in transcriptional regulation and inhibition of apoptosis such as SOX10, FOXC2, JAG1, TCF4, TWIST1, WNT11, SNAI1 and PDGFRB, while reducing the expression of BMP7 and TSPAN13. Together, our results suggest that PRAME holds potential as a biomarker for metastasis in TNBC, and warrant further investigation into PRAME as a therapeutic target.

## Methods

### Cell culture

The triple negative breast cancer cell lines MDA-MB-468 and BT549 were purchased from the American Tissue Culture Collection (ATCC). MDA-MB-468 cells were maintained in Dulbecco’s Modified Eagle’s Medium (Gibco-BRL) supplemented with 10% (v/v) Fetal Bovine Serum (Hyclone US origin, GE Lifescience), 50 U/ml penicillin and 50 μg/ml streptomycin (Gibco-BRL). BT549 cells were maintained in ATCC-formulated Roswell Park Memorial Institute 1640 medium (Gibco-BRL) supplemented with 10% (v/v) Fetal Bovine Serum (Hyclone US origin, GE Lifescience), 50 U/ml penicillin and 50 μg/ml streptomycin (Gibco-BRL), and 0.023 IU/ml insulin (Sigma-Aldrich). All cells lines were maintained in a in a humidified incubator at 37 °C, 5% CO2 and regular mycoplasma testing was performed using a PCR-based assay.

### Transcriptomic analysis of PRAME expression in public datasets

We mined the public data repositories of The Cancer Genome Atlas (TCGA) and the Genotype-Tissue Expression (GTEx) program for transcriptomic data on PRAME in normal and cancerous tissues. We used the Gene Expression Profiling Interactive Analysis (GEPIA) (http://gepia.cancer-pku.cn/index.html) platform for batch data processing and visualization [40]. Box plots were generated to compare PRAME expression between corresponding tumor and normal tissues, and Kaplan–Meier survival curves were obtained for PRAME expression in breast cancer.

### Gene ontology enrichment analysis of differentially expressed EMT-related genes

We conducted enrichment analysis of gene ontology using the Database for Annotation, Visualization and Integrated Discovery (DAVID) (https://david.ncifcrf.gov/tools.jsp). Differentially expressed EMT-related genes were subjected to enrichment analysis for biological process or disease with p ≤ 0.05 and enrichment gene count > 2.

### Stable overexpression of PRAME in MDA-MB-468 cells

Adherent MDA-MB-468 cells were transduced at 80% confluency with purified PRAME lentiviral particles (pReceiver-Lv203, GeneCopoeia) or purified negative control lentiviral particles (LPP-NEG-Lv105-100, GeneCopoeia). After 72 h, transduced MDA-MB-468 cells were maintained under 0.5 µg/ml puromycin (Sigma-Aldrich) selection. Overexpression of PRAME was assessed by real time qRT-PCR, western blot, and immunofluorescence.

### Transient silencing of PRAME in BT549 cells

PRAME siRNA #1–4 smartpool (siGENOME SMARTpool, M-012188-00-0010) and control siRNA #1–4 smartpool (siGENOME Non-Targeting siRNA Pool#1, D-001206-13-20) were purchased from Dharmacon. Adherent cells were transfected at 60–70% confluency with 10 nM siRNA #1–4 and DharmaFect 1 (Dharmacon) following the manufacturer’s instructions. Silencing of PRAME was assessed by real time qRT-PCR, western blot, and immunofluorescence 48–72 h after transfection.

### RNA isolation and cDNA synthesis

Total RNA was isolated using the PureLink RNA Mini kit (Ambion) following the manufacturer’s protocol. The RNA quantity and purity was assessed by Nanodrop measurement. Reverse transcription of 1 µg RNA was performed using MMLV-Superscript and random hexamers resulting in a final concentration of 50 ng/µl cDNA.

### Quantitative real-time RT-PCR

Real time qRT-PCR was conducted using 50 ng cDNA and the cycle conditions were as following: 50 °C for 2 min, then 95 °C for 10 min and followed by 40 amplification cycles (95 °C for 15 s, 60 °C for 1 min). Specific 5′FAM-3′MGB Taqman gene expression primer/probe sets were purchased from Applied Biosystems to determine the mRNA expression of PRAME (Hs01022301_m1), TWIST1 (Hs01675818_s1), ZEB1 (Hs00232783_m1), MMP2 (Hs01548727_m1) & MMP9 (Hs00957562_m1). Expression levels were normalized to the housekeeping gene RPLPO (4333761F).

### EMT RT2 Profiler™ PCR array

Differential expression of EMT-associated genes was analyzed using the EMT RT2 profiler PCR array (Qiagen, PAHS-090) according to the manufacturer’s guidelines, and analysed using the online RT^2^ Profiler PCR Array Data Analysis Tool (Qiagen). Expression was normalized to the housekeeping gene RPLPO, and the threshold value for differential expression was defined as log_2_(FC) ≥ 1.5 or log_2_(FC) ≤  − 1.5.

### Western blotting

Cells were lysed using RIPA lysis buffer (Pierce) containing Complete EDTA-free protease inhibitor cocktail mix (Thermo). Cell lysates were incubated on ice for 15 min, and centrifuged at 14,000 rpm for 15 min. Supernatants were collected and total protein content was determined using the BCA protein assay (Pierce). Protein samples were reduced and denatured in 5× Laemmli sample buffer and equal amounts of protein were loaded onto a 4–15% TGX gel (BioRad). Next, proteins were transferred onto 0.2 µm polyvinylidinedifluoride membranes (BioRad). The membranes were blocked in 5% non-fat dried milk/Tris-buffered saline and 0.1% Tween-20 for 1 h at room temperature, and washed with Tris-buffered saline and 0.1% Tween-20. Next, the membranes were incubated overnight at 4 °C with the following primary antibodies diluted in 5% non-fat dried milk/Tris-buffered saline and 0.1% Tween-20: rabbit anti-PRAME (Thermo, #PA5-1367, 1:500), mouse anti-ECadherin (Abcam clone M168, #ab76055, 1:1000), mouse anti-NCadherin (Cell Signaling technologies clone 13A9, # 14215S, 1:1000), rabbit anti-vimentin (Cell Signaling technologies clone D21H3, #5741, 1:1000), rabbit anti-ZEB1 (Cell Signaling technologies clone D80D3, #3396, 1:1000), rabbit anti-SNAI1 (Cell Signaling technologies clone C15D3, #3879, 1:1000), rabbit anti-TWIST1 (Cell Signaling technologies, #46702, 1:1000) and rabbit anti-βactin (Cell Signaling technologies clone 13E5, #4970, 1:1000). Membranes were washed three times with Tris-buffered saline and 0.1% Tween-20 for 15 min each, probed with horseradish peroxidase-conjugated secondary antibodies (Jackson ImmunoResearch 1:10,000) for 1 h at room temperature and washed four times with Tris-buffered saline and 0.1% Tween-20 for 5 min each. Bound antibodies were detected using ECL Plus or ECL Supersignal-West Femto (Pierce). Densitometry analysis was performed using Fiji software.

### Immunofluorescence

Cells were seeded on Poly-d-Lysine coated coverslips (Corning) and 24 h later fixed with 4% paraformaldehyde (ChemCruz). Cells were permeabilized using phosphate-buffered saline with 1% bovine serum albumin and 0.1% Triton-X100 for 10 min, followed by blocking in phosphate-buffered saline with 10% bovine serum albumin for 30 min. Next, the cells were incubated with primary antibodies for 1 h at room temperature, followed by two wash steps of 30 min each and incubated with Alexa Fluor 488 or Alexa Flour 555-labelled secondary antibodies (Cell Signaling Technologies, 1:500) for 1 h at room temperature. The primary antibodies used were as following; rabbit anti-PRAME (Thermo, #PA5-13679, 1:100), mouse anti-ECadherin (Cell Signaling technologies clone 4A2, #14472, 1:100) and rabbit anti-vimentin (Cell Signaling technologies clone D21H3, #5741, 1:100). Cell nuclei were visualized with DAPI (Thermo) and cells were mounted using Prolong Gold Antifade reagent (Invitrogen). Images were captured using an upright fluorescent microscope (Zeiss Axioimager, 40× objective), equipped with acquisition and analyses software.

### Wound healing assay

Cells were plated in 12 well plates and grown to confluency overnight. Using a p10 pipette tip a scratch was made in each cell monolayer, and the cells were cultured in complete medium for 17 h. For transient silencing of PRAME, the scratch was made 48 h after transfection. Images were taken at 0 h and 17 h after wounding, and analyzed using ImageJ/Fiji software and the MRI wound healing tool. Wound closure at 17 h was defined as a percentage of the original wound area.

### Cell migration assay

Cell migration was performed using the QCM™ 24-well colorimetric cell migration assay (Millipore, Cat. No. ECM508) according to the manufacturer’s protocol. The cells were serum starved for 24 h prior to the assay. Next, cells were seeded in the inserts in FBS-free medium with 5% BSA, and medium with 10% FBS was added to the lower chambers as a chemoattractant. For the BT549 PRAME silenced cell line model, the cells were harvested and seeded on the inserts 48 h after transfection. Finally, the cells were allowed to migrate for 24 h, after which the non-migrated cells were removed with a cotton swab, migrated cells were stained, pictures of the stained inserts were taken and the rate of cell migration was determined by colorimetric measurement at 560 nm.

### 3D inverted invasion assay

Cell invasive capabilities were assessed using a 3D inverted invasion assay. A suspension of 5–6 mg/ml Matrigel or 2 mg/ml collagen I (First Link) was transferred to 8 µm pore Transwell™ inserts (Corning) to polymerise at 37 °C, 5% CO_2_. Next, inserts were inverted and cells were seeded on the underside of the membrane, left to adhere, followed by gentle washing to remove non-adherent cells. The transwell inserts were placed into a clean 24-well plate containing serum-free medium, medium supplemented with 10% FBS was added on top of the matrix to provide a chemotactic gradient and cells were allowed to invade for 72 h. Cells were stained with 4 µM Calcein-AM (Sigma-Aldrich) and visualised by confocal microscopy. For each transwell, 3 fields of view were selected and serial optical sections were imaged at 15 µm intervals giving a total of 9 fields of view for each experimental condition. The rate of invasion was quantified using ImageJ/Fiji and the Area Calculator plugin as the percentage of invasion beyond 30 μm compared to the total fluorescence intensity of all cells within the gel.

### Matrigel Boyden Chamber invasion assay

Cell invasion through Matrigel was further investigated using the 8 µm QCM ECMatrix Cell Invasion Assay (Millipore, Cat. No. ECM550). Cells were seeded in serum-free medium in the top chamber, and medium supplemented with 10% FBS was added to the bottom chamber. Cells were allowed to invade through the Matrigel-coated membrane for 72 h, followed by staining with crystal violet. Images of the stained inserts were taken and the rate of invasion was determined by colorimetric measurement at 560 nm.

### Statistics

Data were analyzed using the 2-tailed unpaired t test and are represented as mean ± SEM unless stated otherwise. Normality of data was assessed using the D’Agostino-Pearson normality test.

## Results

### PRAME expression in breast cancer

**Fig. 1:**
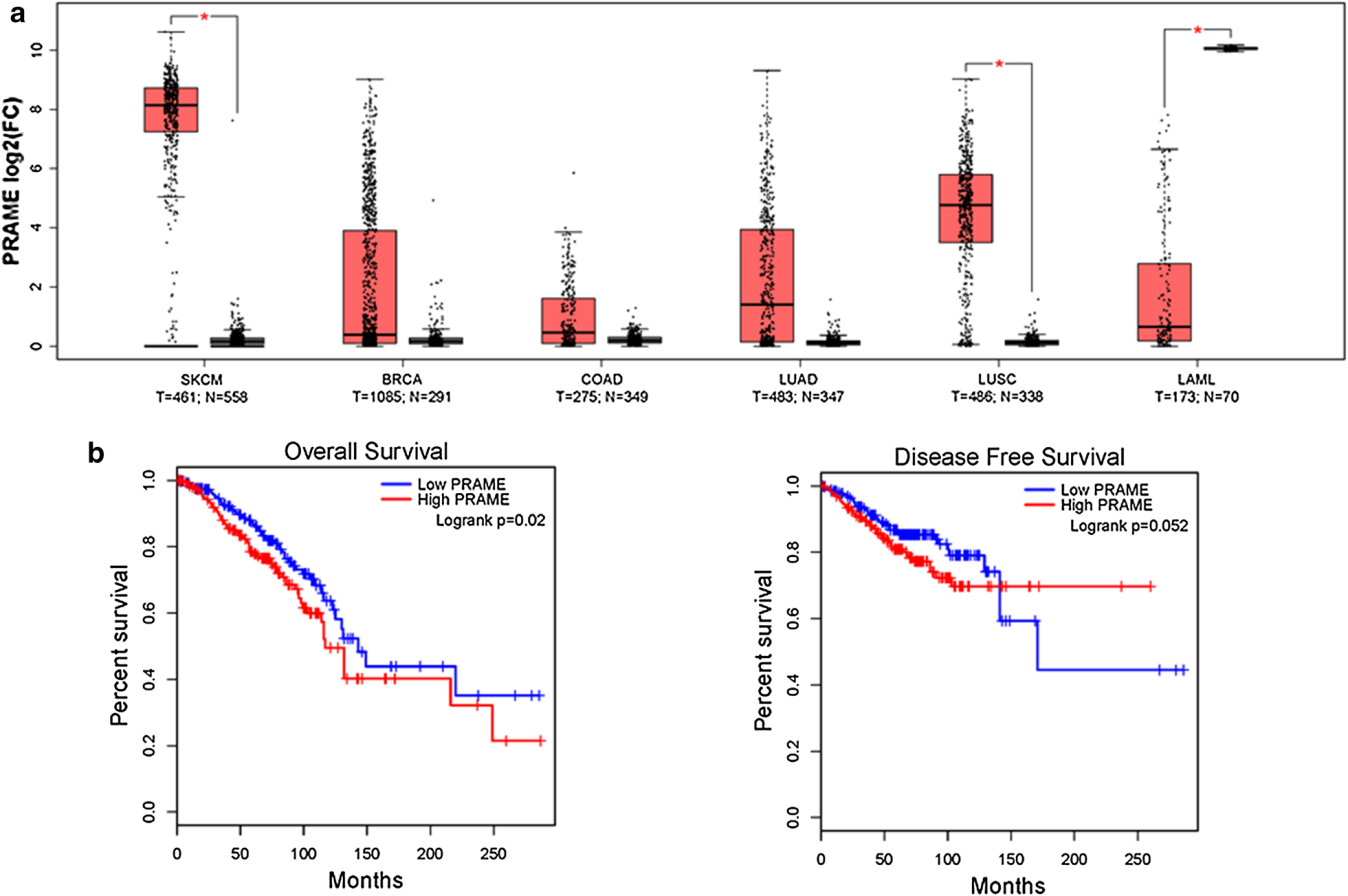
PRAME expression in common malignancies and association with survival in breast cancer. **a** *PRAME* mRNA expression across the most common human malignancies comparing expression in tumor (T, red) and normal (N, grey) tissue. Boxplot represents Median and IQR. *p ≤ 0.05, One-way ANOVA with log_2_FC cutoff ≥ 1.5 or ≤ − 1.5. SKCM, Skin Cutaneous Melanoma; BRCA, breast invasive carcinoma; COAD, colon adenocarcinoma; LUAD, lung adenocarcinoma; LUSC, lung squamous cell carcinoma; LAML, acute myeloid leukemia. **b** Kaplan–Meier curves for overall and disease-free survival of *PRAME* expression in breast cancer. Patients were classified into subgroups with low (n = 531) or high (n = 534) expression defined as lower or as higher than median *PRAME* mRNA expression. p-value obtained by log-rank test

We interrogated the TCGA pan-cancer and GTEx data repositories for *PRAME* mRNA expression using the Gene Expression Profiling Interactive Analysis (GEPIA) interactive web server [40]. We found an increased expression of *PRAME* in cancer tissues compared to the corresponding normal tissues for the most common cancer types among women worldwide (breast cancer, colorectal cancer, lung cancer) in addition to its well-known upregulation in melanoma (Fig. 1a). In contrast, *PRAME* expression was downregulated in acute myeloid leukemia, where it has been reported to be associated with a more favorable outcome. Next, we explored the association of *PRAME* with clinical outcome in the TCGA breast cancer dataset and found that *PRAME* expression correlated significantly with a shorter overall survival and to a lesser extent with a shorter disease-free survival (Fig. 1b).

### PRAME cell line models

**Fig. 2:**
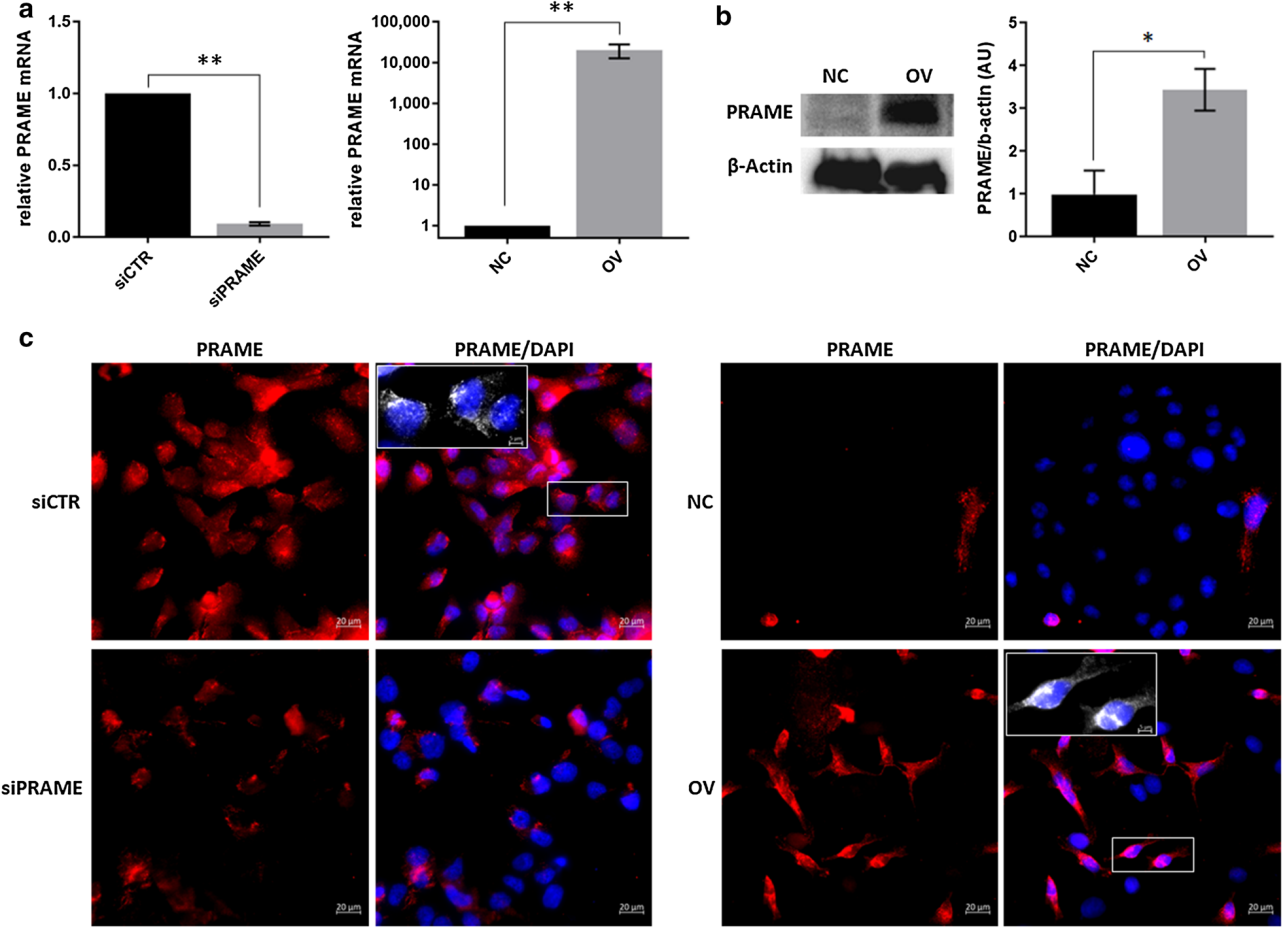
PRAME mRNA and protein expression in silenced and overexpressing TNBC cell line models. **a** Relative *PRAME* mRNA expression, normalized to the housekeeping gene RPLPO. **b** Representative picture and densitometric quantification of PRAME protein expression, as determined by western blot. *p ≤ 0.05. **c** Representative immunofluorescence pictures of PRAME localization. Magnification ×40. DAPI, blue; PRAME, red. Insert at 130% zoom

In order to study the role of PRAME in triple negative breast cancer, PRAME expression was manipulated by transient silencing in BT549 cells and stable overexpression in MDA-MB-468 cells. PRAME expression was significantly decreased after silencing, and increased after transduction with the PRAME-GFP lentiviral vector as confirmed by qPCR and western blotting (Fig. 2a, b). Using immunofluorescence, we could detect PRAME expression in both the nucleus and cytoplasm of both cell line models (Fig. 2c).

### PRAME induces migration of TNBC cells

**Fig. 3:**
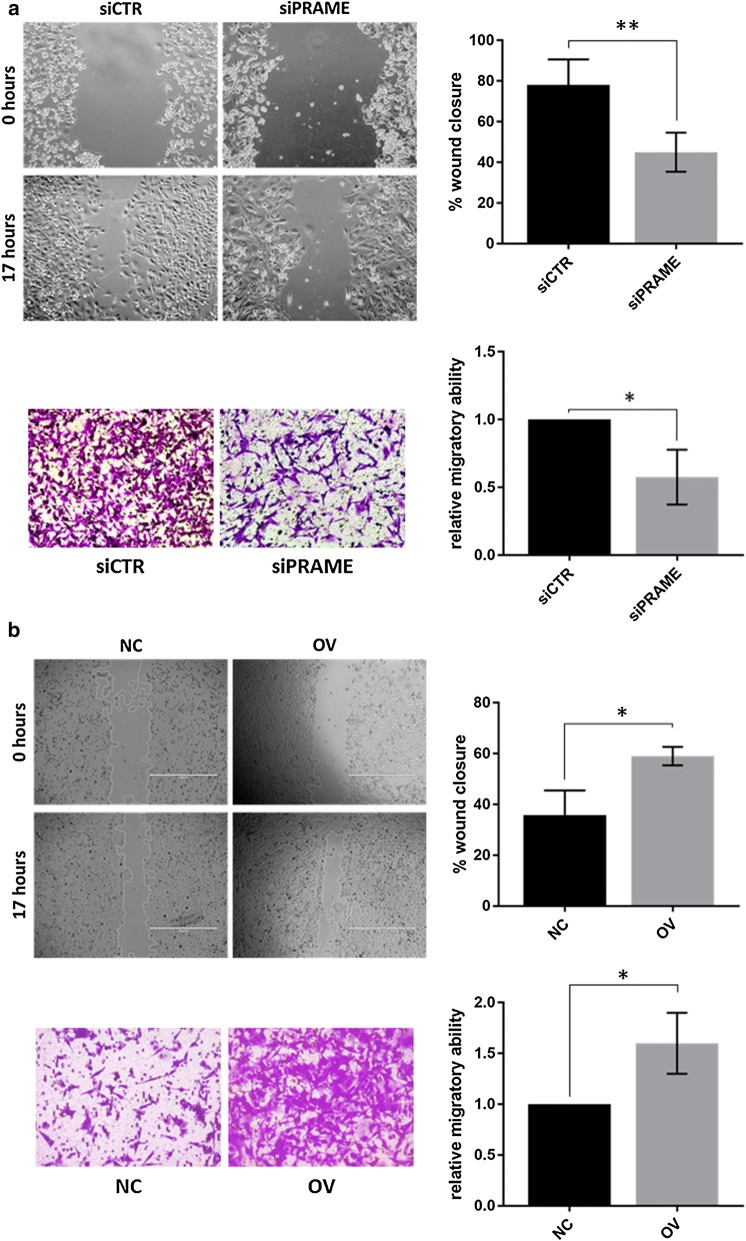
PRAME alters migratory potential of triple negative breast cancer cells. Migratory ability of **a** TNBC cells treated with siCTR or siPRAME for 72 h or **b** TNBC cells stably transduced with control vector or a vector encoding full length PRAME. Migration potential was measured by both the wound closure assay and the QCM™ 24-well colorimetric cell migration assay (Boyden chamber principle). **p < 0.01, *p < 0.05, n = 3 biological replicates

The reportedly ambiguous role of PRAME in the migratory behavior of cancer cells was assessed in triple negative breast cancer using 2 different methodologies; the wound healing assay and a Boyden chamber migration assay. Using both techniques, we found that silencing of PRAME reduced tumor cell migration by an average of 40% (Fig. 3a), while overexpression of PRAME increased the migratory potential of TNBC cells by an average of 60% (Fig. 3b).

### PRAME facilitates invasion of TNBC cells

**Fig. 4:**
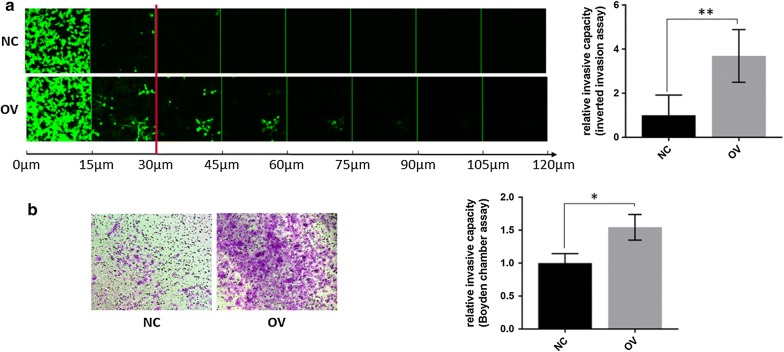
PRAME overexpression mediates invasion through 3D Matrigel. **a** Inverted Matrigel invasion of TNBC cells stably transduced with control vector or full-length PRAME. Live cells were visualized using Calcein-AM and serial optical sections were acquired at 15 μm intervals, with increasing depth from left to right. Rate of invasion beyond 30 μm, indicated by red line, was quantified in 3 fields of view/biological replicate (n = 3). **p < 0.01. **b** Matrigel Boyden Chamber invasion assay of TNBC cells stably transduced with control vector or full-length PRAME, performed in QCM ECMatrix Cell Invasion Assay. *p < 0.05, n = 3 biological replicates

We interrogated the role of PRAME in invasion as an in vitro model for metastasis. Similar to the migration analyses, we utilized two different methodologies to assess the invasive potential of TNBC cells. Using a 3D inverted invasion assay as well as a Boyden chamber invasion assay, we found that PRAME overexpression increases the invasion of TNBC cells through Matrigel, a model for extravasation into the blood circulation (Fig. 4). More prominent differences in Matrigel invasion were observed using the inverted invasion assay, supporting the role of active invasion of individual cancer cells. We did not observe any significant differences in invasion through collagen type I, suggesting that PRAME might not be involved in local invasion of TNBC (data not shown). Single and EMT array qPCR analysis of the matrix metalloproteinases MMP2 and MMP9, key mediators of blood vessel basement membrane degradation and metastasis, revealed an average threefold upregulation of *MMP2* mRNA expression but not of *MMP9* (data not shown). Further analysis determining the enzyme activity of MMP2 and MMP9 are currently undertaken.

### Silencing of PRAME attenuates the mesenchymal phenotype of TNBC cells

**Fig. 5:**
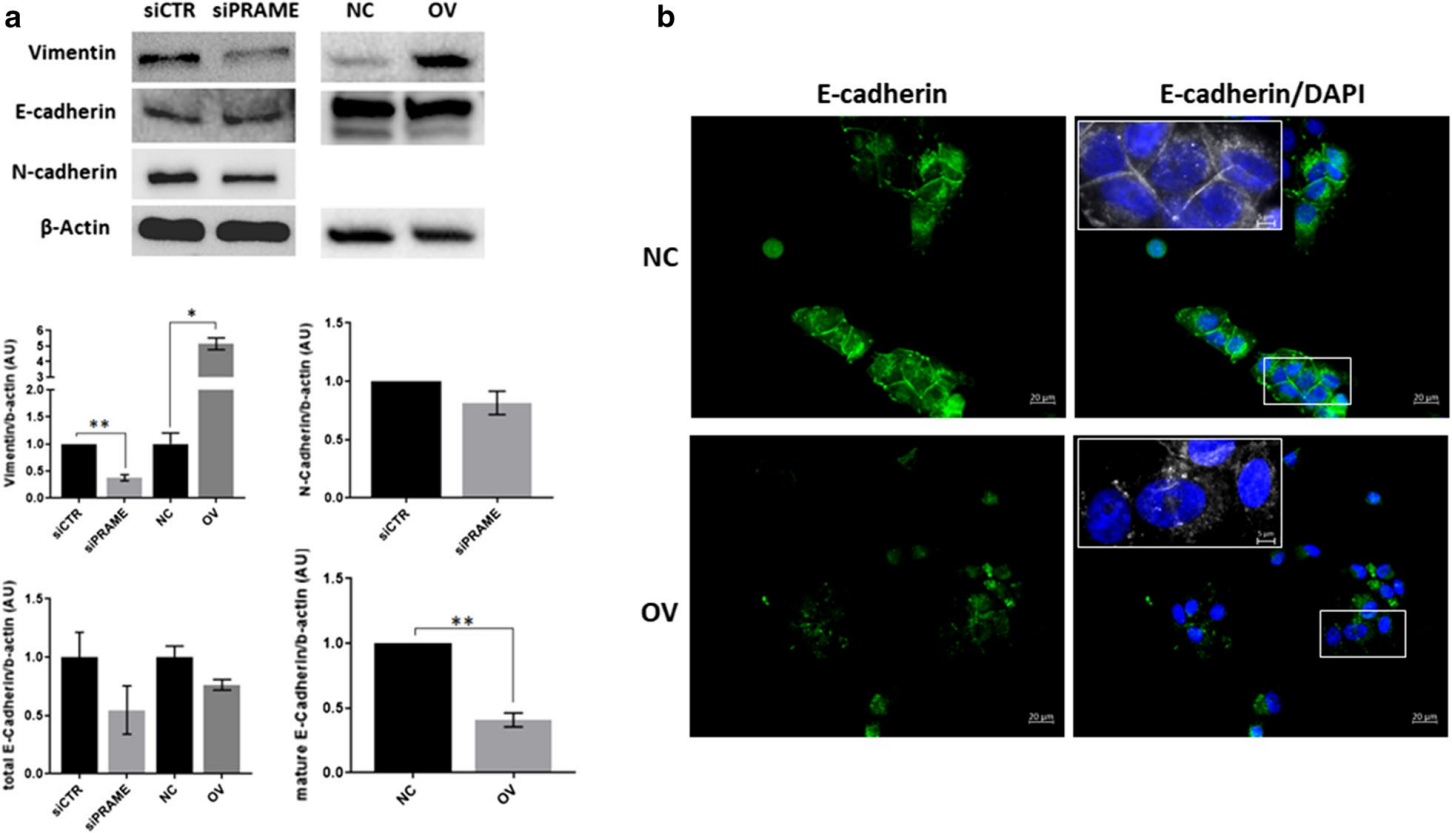
PRAME induces an epithelial-to-mesenchymal transition towards a more mesenchymal phenotype. **a** Protein expression and densitometric quantification of epithelial (E-Cadherin) and mesenchymal (Vimentin, N-Cadherin) markers in PRAME TNBC cell line models. N-Cadherin expression could not be detected in the MDA-MB-468 cell line. *p ≤ 0.05; **p ≤ 0.01. **b** Representative **i**mmunofluorescence image of E-cadherin, demonstrating subcellular redistribution in PRAME overexpressing cells. Magnification ×40. DAPI, blue; E-Cadherin, green. Insert at 130% zoom

Given the inherent mesenchymal phenotype of triple negative breast cancer cells, we determined the expression of the mesenchymal marker vimentin and the epithelial marker E-cadherin in relation to PRAME expression (Fig. 5a). We found that vimentin protein expression was significantly reduced after silencing of PRAME, and increased upon PRAME overexpression. Silencing of PRAME in BT549 cells induced cadherin switching, as demonstrated by a decrease in N-cadherin expression in the absence of significant changes in E-cadherin expression. Interestingly, overexpression of PRAME in MDA-MB-468 cells significantly reduced the levels of mature E-cadherin and induced a redistribution of E-cadherin from the cell surface to punctate perinuclear and cytosolic structures (Fig. 5b). N-cadherin protein expression of MDA-MB-468 cells was too low to be detected, although we observed a twofold upregulation in mRNA expression after PRAME overexpression using the EMT RT^2^ Profiler PCR Array (data not shown). Concomitantly, PRAME overexpression induced a 1.7-fold increase in fibronectin mRNA expression (data not shown).

### PRAME regulates the expression of multiple EMT-related genes

**Fig. 6:**
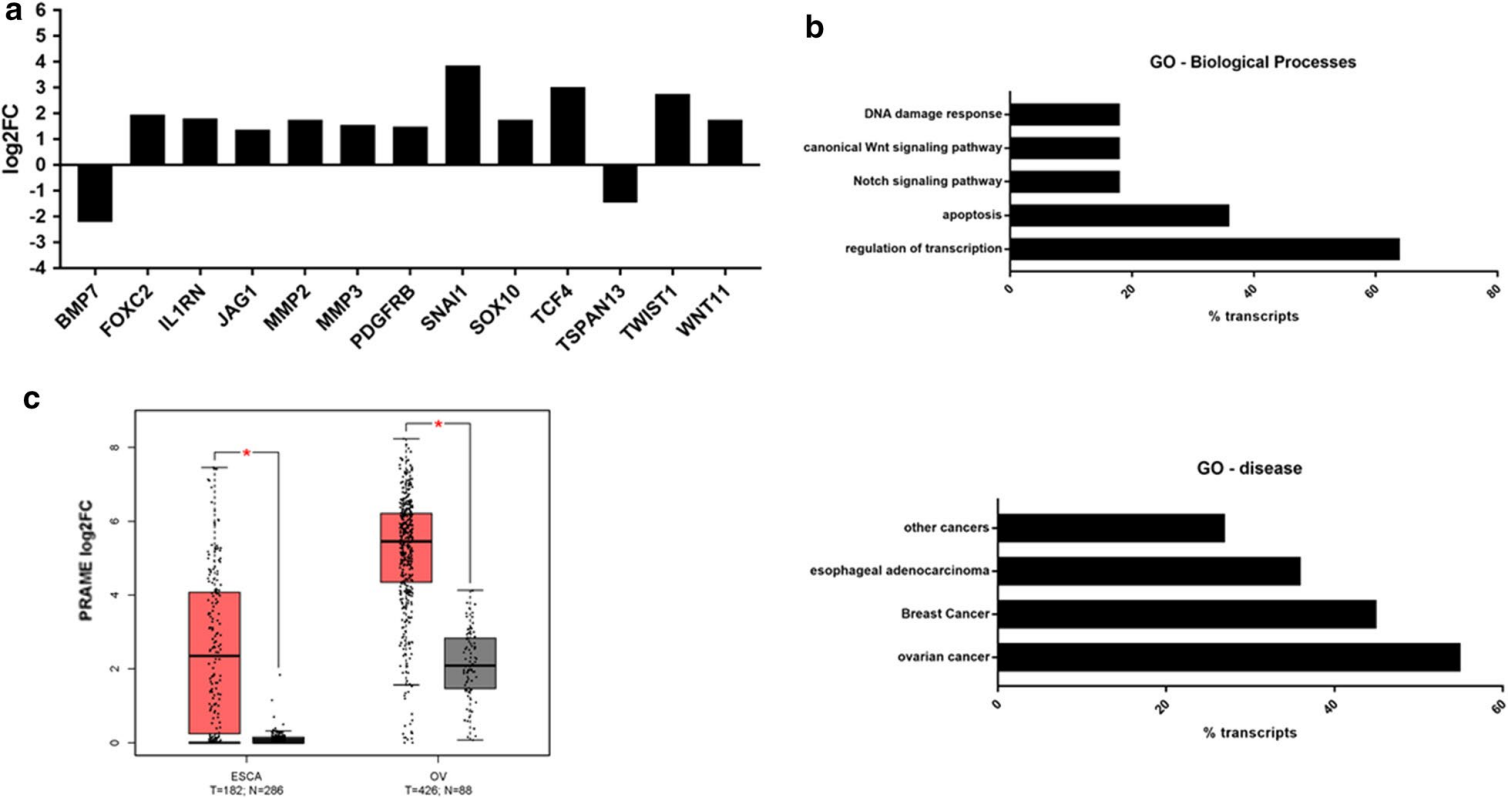
PRAME plays a role in gene expression reprogramming during epithelial-to-mesenchymal transition. **a** PRAME overexpression results in upregulation of 11 and downregulation of 2 EMT-related genes as determined by the EMT RT2 Profiler qPCR assay. **b** Gene ontology analyses of PRAME-associated upregulated genes according to enrichment for biological processes and disease-inference. **c** TCGA and GTEx data analysis of *PRAME* mRNA expression in esophageal and ovarian cancer using the GEPIA web server. T, tumor (red); N, normal (gray). Boxplot represents median and IQR. *p < 0.05, One-way ANOVA with log_2_FC cutoff ≥ 1.5 or ≤ − 1.5

In order to better understand how PRAME can alter the migratory behavior and mesenchymal phenotype of TNBC cells, we investigated whether PRAME is involved in the regulation of EMT-related genes. Using the EMT RT^2^ Profiler PCR Array, we determined the mRNA expression levels of 84 key genes that are altered during EMT; including signal transduction pathway molecules and genes involved in differentiation and development, cell morphogenesis, cell growth and proliferation, cell migration and motility, cytoskeleton regulation, and adhesion. Using a threshold of log_2_FC ≥ 1.5 or log_2_FC ≤ − 1.5, overexpression of PRAME induced the expression of 11 EMT-related genes (*SNAI1*, *TCF4*, *TWIST1*, *FOXC2*, *IL1RN*, *MMP2*, *SOX10*, *WNT11*, *MMP3*, *PDGFRB*, *JAG1*), while reducing the expression of 2 EMT-related genes (*BMP7* and *TSPAN13*) (Fig. 6a). Gene ontology enrichment analyses revealed that the upregulated genes are not only involved in promoting epithelial-to-mesenchymal transition and cell migration but also play a role in transcriptional regulation and inhibition of apoptosis (Fig. 6b). Many of the upregulated genes encode for transcription factors that play a role in Notch and Wnt signaling, which are often dysregulated in cancer. GO-disease inference revealed frequent aberration of the PRAME-differentially expressed genes in ovarian and esophageal cancer, suggesting that PRAME and its effectors may also play a role in these solid cancer types. Indeed, further analyses of TCGA and GTEx data showed that PRAME expression is also upregulated in ovarian and esophageal cancer tissues compared to their normal counterparts (Fig. 6c).

### PRAME alters the expression of EMT-transcription factors

**Fig. 7:**
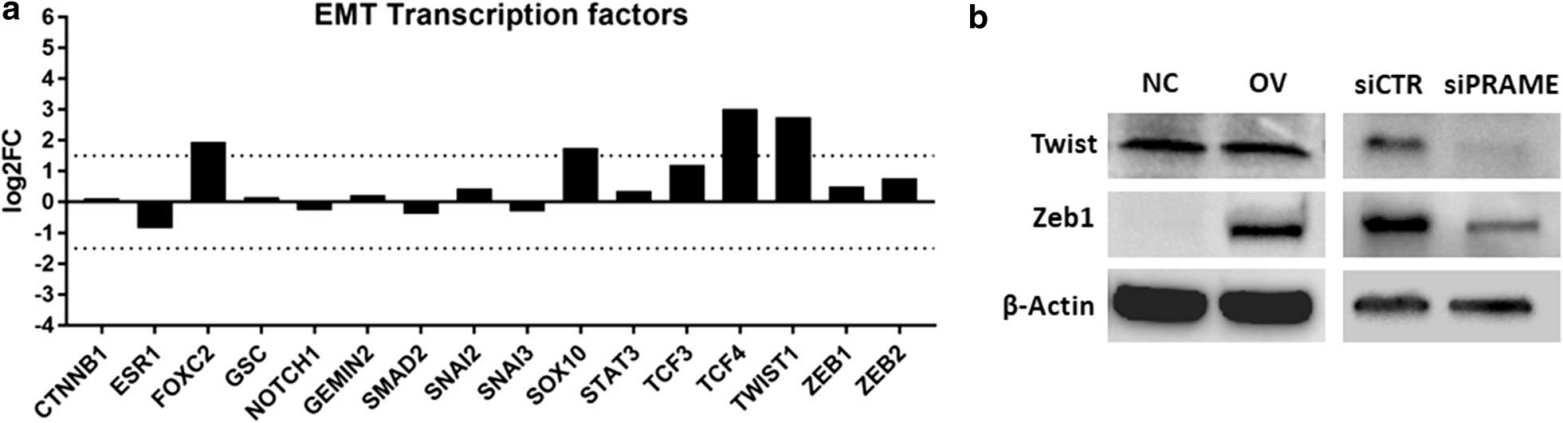
PRAME induces the expression of several epithelial-to-mesenchymal transition transcription factors. **a** PRAME overexpression results in upregulation of 4 EMT-transcription factors as determined by the EMT RT2 Profiler qPCR assay. **b** Protein expression of TWIST1 and ZEB1 in PRAME TNBC cell line models

Next, we investigated whether PRAME can induce EMT by regulating the expression of EMT transcription factors. RNA expression of 16 EMT-specific TFs revealed that overexpression of PRAME induced the RNA expression of *TCF4*, *TWIST1*, *FOXC2*, *SOX10*, *TCF3*, *ZEB2*, *ZEB1*, *SNAI2*, *STAT3*, *GEMIN2* and *GSC* (Fig. 7a). Out of 11 EMT TFs; *FOXC2*, *SOX10*, *TCF4* and *TWIST1* showed an increase in expression of minimum 2.5-fold or log_2_FC ≥ 1.5. Since TWIST1 together with ZEB1 are reported to be the core EMT-driving factors, we confirmed changes in mRNA expression by single qRT-PCR assays (data not shown) and further explored their expression patterns at the protein level. We found that PRAME overexpression increased protein expression of ZEB1 whereas silencing of PRAME reduced the protein expression of both ZEB1 and TWIST1 (Fig. 7b). Despite a sixfold increase in *TWIST1* mRNA expression after PRAME overexpression, we could not detect any changes in TWIST1 protein levels.

## Discussion

PRAME re-expression has been demonstrated in several solid and hematological cancers, driving clinical trials investigating PRAME as an immunotherapeutic target. However, the biological function and molecular mechanisms of PRAME are not completely elucidated. Compelling evidence exists on its role as a dominant repressor of retinoic acid receptor signaling thereby inhibiting retinoic acid-induced differentiation, cell cycle arrest and apoptosis [11, 33]. Further, PRAME has been shown to increase cell proliferation and inhibit apoptosis in various cancer models [34–36, 41]. However, the role of PRAME in migration and invasion—an important hallmark of cancer progression—remains elusive. In the present study, we provide experimental evidence that PRAME exerts its tumor-promoting function in part by increasing the cancer cell’s motility, hence possibly enhancing its metastatic abilities. In line with this, we and others have demonstrated that PRAME expression is associated with advanced disease and poor disease-free and overall survival.

Using several approaches, we show here that PRAME expression increases the cell motility of triple negative breast cancer cells. PRAME overexpression significantly increased both cell migration and invasion, while silencing of PRAME markedly reduced cell migration. Next, we sought to evaluate whether these changes in migratory behaviour were induced by an epithelial-to-mesenchymal transition. Overexpression of PRAME increased the expression of the mesenchymal marker vimentin and induced a re-distribution of the epithelial marker E-cadherin from the cell surface to punctate cytosolic structures. Silencing of PRAME on the other hand reduced vimentin expression and induced a cadherin switch with a reduction in N-cadherin expression. Together, these findings suggest that PRAME is involved in promoting EMT towards a more motile, mesenchymal phenotype. This is in stark contrast to the sole 2 studies to date exploring the role of PRAME in EMT and invasion [38, 39]. In those 2 studies, silencing of PRAME reduced E-cadherin expression and promoted invasion in addition to increasing cell proliferation and inhibiting apoptosis. The discordance between our findings could in part be caused by the use of different cell lines, representing distinct cancer types and TNBC (sub)types which are associated with different gene expression patterns and clinical outcome [7, 8]. Our study focused on triple negative breast cancer cell lines from the basal-like (MDA-MB-468) and mesenchymal (BT549) subtype, whereas the studies by Xiao J et al. investigated 1 TNBC cell line of the mesenchymal stem-like (MDA-MB-231) subtype in addition to a non-TNBC cell line (MCF7) and 2 lung cancer cell lines (PC9 and A549). Differences in our findings may also be explained partly by variations in methodology. While we assessed changes in cell motility and EMT after 48–72 h, they determined the rate of invasion 24 h after silencing which could lead to different results due to temporal variations in cell motility dynamics. Furthermore, differences in experimental design could also contribute to the contradictory increase in cell proliferation and inhibition of apoptosis they observed after PRAME knockdown as compared to what has been reported by others [34–36].

In order to validate our findings and to gain insight into the molecular mechanisms underlying the EMT induction by PRAME, we performed an in-depth analysis of 84 EMT-related genes. We found that PRAME overexpression in triple negative breast cancer cells resulted in an upregulation of 11 genes (*SNAI1*, *TCF4*, *TWIST1*, *FOXC2*, *IL1RN*, *MMP2*, *SOX10*, *WNT11*, *MMP3*, *PDGFRB*, *JAG1*) and a downregulation of 2 genes (*BMP7* and *TSPAN13*). Gene ontology revealed that many of the upregulated genes encode for proteins that are additionally involved in transcriptional regulation and inhibition of apoptosis. Furthermore, disease inference showed that several of the PRAME-differentially expressed genes are frequently dysregulated in ovarian and esophageal cancer. Thus, we explored the expression of PRAME in both solid cancer types using the TCGA data repository and found a significant increase in *PRAME* expression in the tumor tissues compared to normal tissues. Among the PRAME-associated upregulated genes, we identified the established EMT transcription factors *SNAI1* and *TWIST1* as well as several newly implicated factors. SNAI1, TWIST1 and ZEB1 are the master regulators of the epithelial-to-mesenchymal transition that repress the expression of E-cadherin, and activate genes that contribute to the mesenchymal phenotype including N-cadherin, MMP2, MMP9 and each other [42]. We were able to demonstrate that PRAME manipulation alters the RNA expression of all 3 regulators, and in addition strongly affects the protein expression of ZEB1. More recently, additional transcription factor families have been identified as regulators of gene expression reprogramming during EMT, including the forkhead box (FOX), SRY box (SOX) and T-cell factors (TCF) transcription factors. In accordance, we observed an upregulation of *FOXC2*, *SOX10*, and *TCF4* in addition to *Wnt11* and *JAG1* after PRAME overexpression. These transcription factors are key players in the Notch and Wnt signaling pathways which are often dysregulated in cancer; thereby regulating stem cell maintenance, cell differentiation and growth [43, 44]. FOXC2 expression is upregulated during EMT by SNAI1 and TWIST1, and enhances migration and invasion by increasing the expression of the mesenchymal markers vimentin and N-cadherin [45]. Interestingly, unlike SNAI1 and TWIST1, FOXC2 did not repress E-cadherin expression in canine kidney epithelial cells but redistributed E-cadherin from the cell surface to the cytosol. These findings are in line with our observations that PRAME overexpression redirects E-cadherin expression to punctate cytosolic structures. In addition, JAG1 and SOX10 can induce mesenchymal features, and have been found to promote the migratory and invasive abilities of breast cancer cells [43, 46]. Of note, both molecules have been found at significant higher levels in the aggressive, metastatic triple negative breast cancer subtype compared to other subtypes [47, 48]. This supports our hypothesis that PRAME, and its network, might play an important role in the acquisition of cellular traits that confer the aggressive behavior of triple negative breast cancer. As such, PRAME harbors potential as a prognostic biomarker or therapeutic target for this specific subgroup of breast cancer patients. Furthermore, upregulation of *TCF4* could partly explain the observed PRAME-mediated EMT through increased activation of the TCF4/β-catenin transcriptional complex resulting in increased expression of ZEB1, followed by reduced E-Cadherin expression and increased vimentin expression [49]. We observed an upregulation of *Wnt11* that previously has been shown to increase cell proliferation, migration and invasion in breast cancer [50]. Together, our data suggest that select transcription factors may be downstream effectors of PRAME, contributing to the acquisition of mesenchymal traits. In addition to an upregulation of transcription factors, we found an increased expression of *IL1RN* and the matrix metalloproteinases *MMP2* and *MMP3* in PRAME overexpressing breast cancer cells. IL1RN encodes the endogenous IL-1 receptor antagonist that binds the IL-1 receptor, thereby modulating the activity of the pro-inflammatory cytokine IL-1. Upregulation of IL1RN has been demonstrated in various tumor types in association with increased cell proliferation and a higher risk of metastasis [51, 52]. Increased expression of MMP2 and MMP3 could further propagate a more motile mesenchymal phenotype through degradation of the extracellular matrix, cleavage of cell surface E-cadherin and indirect transcriptional activation of Snail [53].

Mechanistic analyses revealed a downregulation of *BMP7* and *TSPAN13* after overexpression of PRAME. Previous studies have demonstrated an important role for BMP7 in the maintenance of an epithelial phenotype by induction of MET and inhibition of TGFβ1-mediated cell proliferation and metastasis [54]. Furthermore, reduced expression of BMP7 in breast cancer has been correlated with increased risk of specifically bone metastases [55]. TSPAN13 expression is significantly reduced in estrogen negative, Her2 negative and basal-like breast tumors [56]. Ectopic expression of TSPAN13 in breast cancer cells has been reported to inhibit anchorage independent growth, increase apoptosis and reduce invasion [57]. Thus, the downregulation of both *BMP7* and *TSPAN13* in conjunction with the upregulation of aforementioned genes strongly supports a tumor-promoting role for PRAME.

## Conclusions

We demonstrate that PRAME facilitates the transition to a mesenchymal phenotype through reprogramming of several EMT-genes, resulting in enhanced migration and invasion of triple negative breast cancer cells. Moreover, increased PRAME expression was correlated with a worse survival, further supporting its clinical value as a prognostic biomarker and/or therapeutic target in cancer.

## Abbreviations

5-azaC: 5-aza-2′-deoxycytidine
BMP7: bone morphogenetic protein 7
CT130: cancer testis antigen 130
CTA: cancer testis antigen
EMT: epithelial-to-mesenchymal transition
EGFR: epidermal growth factor receptor
ER: estrogen receptor
FOXC2: forkhead box C2
MFH-1: mesenchyme forkhead 1
GEMIN2: Gem Nuclear Organelle Associated Protein 2
GEPIA: gene expression Profiling interactive analysis
GO: gene ontology
GSC: Goosecoid homeobox
GTEx: Genotype-Tissue Expression
Her2: human epidermal growth factor receptor 2
IL-1: Interleukin 1
IL1RN: Interleukin 1 receptor antagonist
JAG1: Jagged 1
MAPE: melanoma antigen preferentially expressed in tumors
MMP2: matrix metallopeptidase 2
MMP3: matrix metallopeptidase 3
MMP9: matrix metalloproteinase 9
MZF1: myeloid zinc finger 1
OIP4: OPA-interacting protein 4
PR: progesterone receptor
PARP: Poly ADP-ribose Polymerase
PDGFRB: platelet-derived growth factor receptor, beta polypeptide
PRAME: PReferentially Antigen expressed in Melanoma
RPLPO: ribosomal protein lateral stalk subunit P0
SNAI1: Snail homolog 1
SNAI2: Snail Family Transcriptional Repressor 2
SOX10: SRY (sex determining region Y)-box 10
STAT3: signal transducer and activator of transcription 3
TCF3: transcription factor 3
TCF4: transcription factor 4
TCGA: The Cancer Genome Atlas
TGFβ1: transforming growth factor beta1
TNBC: triple negative breast cancer
TSPAN13: tetraspanin 13
TWIST1: Twist homolog 1
Wnt11: Wingless-type MMTV integration site family, member 11
ZEB1: zinc finger E-box-binding homeobox 1
ZEB2: zinc finger E-box Binding Homeobox 2

## References

[1] World Health Organization (2003). World Cancer Report 2014; International Agency for Research on Cancers.

[2] Perou CM, Sørlie T, Eisen MB, Van De Rijn M, Jeffrey SS, Rees CA, Pollack JR, Ross DT, Johnsen H, Akslen LA (2000). Molecular portraits of human breast tumours. Nature.

[3] Sørlie T, Perou CM, Tibshirani R, Aas T, Geisler S, Johnsen H, Hastie T, Eisen MB, Van De Rijn M, Jeffrey SS, Thorsen T (2001). Gene expression patterns of breast carcinomas distinguish tumor subclasses with clinical implications. Proc Natl Acad Sci USA.

[4] Bray F, Ren JS, Masuyer E, Ferlay J (2013). Estimates of global cancer prevalence for 27 sites in the adult population in 2008. Int J Cancer.

[5] Yuan N, Meng M, Liu C, Feng L, Hou L, Ning Q, Xin G, Pei L, Gu S, Li X (2014). Clinical characteristics and prognostic analysis of triple-negative breast cancer patients. Mol Clin Oncol..

[6] Tong CWS, Wu M, Cho WCS, To KKW (2018). Recent advances in the treatment of breast cancer. Front Oncol.

[7] Lehmann BD, Bauer JA, Chen X, Sanders ME, Chakravarthy AB, Shyr Y, Pietenpol JA (2011). Identification of human triple-negative breast cancer subtypes and preclinical models for selection of targeted therapies. J Clin Invest..

[8] Masuda H, Baggerly KA, Wang Y, Zhang Y, Gonzalez-Angulo AM, Meric-Bernstam F, Valero V, Lehmann BD, Pietenpol JA, Hortobagyi GN (2013). Differential response to neoadjuvant chemotherapy among 7 triple-negative breast cancer molecular subtypes. Clin Cancer Res.

[9] Schenk T, Stengel S, Goellner S, Steinbach D, Saluz HP (2007). Hypomethylation of PRAME is responsible for its aberrant overexpression in human malignancies. Genes Chromosomes Cancer.

[10] Ikeda H, Lethé B, Lehmann F, van Baren N, Baurain JF, de Smet C, Chambost H, Vitale M, Moretta A, Boon T (1997). Characterization of an antigen that is recognized on a melanoma showing partial HLA loss by CTL expressing an NK inhibitory receptor. Immunity.

[11] Epping MT, Bernards R (2006). A causal role for the human tumor antigen preferentially expressed antigen of melanoma in cancer. Cancer Res.

[12] Wadelin F, Fulton J, McEwan PA, Spriggs KA, Emsley J, Heery DM (2010). Leucine-rich repeat protein PRAME: expression, potential functions and clinical implications for leukaemia. Mol Cancer.

[13] Roszik J, Wang W-L, Livingston JA, Roland CL, Ravi V, Yee C, Hwu P, Futreal A, Lazar AJ, Patel SR (2017). Overexpressed PRAME is a potential immunotherapy target in sarcoma subtypes. Clin Sarcoma Res..

[14] Oyama K, Kanki K, Shimizu H, Kono Y, Azumi J, Toriguchi K, Hatano E, Shiota G (2017). Impact of preferentially expressed antigen of melanoma on the prognosis of Hepatocellular Carcinoma. Gastrointest Tumors.

[15] Thongprasert S, Yang P-C, Lee JS, Soo R, Gruselle O, Myo A, Louahed J, Lehmann FF, Brichard VG, Coche T (2016). The prevalence of expression of MAGE-A3 and PRAME tumor antigens in East and South East Asian non-small cell lung cancer patients. Lung Cancer.

[16] Warnecke-Eberz U, Metzger R, Hölscher AH, Drebber U, Bollschweiler E (2016). Diagnostic marker signature for esophageal cancer from transcriptome analysis. Tumour Biol.

[17] Lerut E, Van Poppel H, Joniau S, Gruselle O, Coche T, Therasse P (2015). Rates of MAGE-A3 and PRAME expressing tumors in FFPE tissue specimens from bladder cancer patients: potential targets for antigen-specific cancer immunotherapeutics. Int J Clin Exp Pathol..

[18] Szczepanski MJ, Whiteside TL (2013). Elevated PRAME expression: what does this mean for treatment of head and neck squamous cell carcinoma?. Biomark Med..

[19] Thomas R, Al-Khadairi G, Roelands J, Hendrickx W, Dermime S, Bedognetti D, Decock J (2018). NY-ESO-1 based immunotherapy of cancer: current perspectives. Front Immunol..

[20] Liu Z, Li M, Jiang Z, Wang X (2018). A comprehensive immunologic portrait of triple-negative breast cancer. Transl Oncol..

[21] Ortmann CA, Eisele L, Nückel H, Klein-Hitpass L, Führer A, Dührsen U, Zeschnigk M (2008). Aberrant hypomethylation of the cancer—testis antigen PRAME correlates with PRAME expression in acute myeloid leukemia. Ann Hematol.

[22] Gutierrez-Cosío S, de la Rica L, Ballestar E, Santamaría C, Sánchez-Abarca LI, Caballero-Velazquez T, Blanco B, Calderón C, Herrero-Sánchez C, Carrancio S (2012). Epigenetic regulation of PRAME in acute myeloid leukemia is different compared to CD34 + cells from healthy donors: effect of 5-AZA treatment. Leuk Res.

[23] Siebenkäs C, Chiappinelli KB, Guzzetta AA, Sharma A, Jeschke J, Vatapalli R, Baylin SB, Ahuja N (2017). Inhibiting DNA methylation activates cancer testis antigens and expression of the antigen processing and presentation machinery in colon and ovarian cancer cells. PLoS ONE.

[24] Lee Y-K, Park U-H, Kim E-J, Hwang J-T, Jeong J-C, Um S-J (2017). Tumor antigen PRAME is up-regulated by MZF1 in cooperation with DNA hypomethylation in melanoma cells. Cancer Lett.

[25] Doolan P, Clynes M, Kennedy S, Mehta JP, Crown J, O’Driscoll L (2008). Prevalence and prognostic and predictive relevance of PRAME in breast cancer. Breast Cancer Res Treat.

[26] Epping MT, Hart AA, Glas AM, Krijgsman O, Bernards R (2008). PRAME expression and clinical outcome of breast cancer. Br J Cancer.

[27] Gezgin G, Luk SJ, Cao J, Dogrusöz M, van der Steen DM, Hagedoorn RS, Krijgsman D, van der Velden PA, Field MG, Luyten GPM (2017). PRAME as a potential target for immunotherapy in metastatic uveal melanoma. JAMA Ophthalmol..

[28] Greiner J, Schmitt M, Li L, Giannopoulos K, Bosch K, Schmitt A, Dohner K, Schlenk RF, Pollack JR, Dohner H (2006). Expression of tumor-associated antigens in acute myeloid leukemia: implications for specific immunotherapeutic approaches. Blood.

[29] Abdelmalak CA, Yahya RS, Elghannam DM, El-Khadragy AE, Abd-El-Messih HM (2014). PRAME gene expression in childhood acute lymphoblastic leukemia: impact on prognosis. Clin Lab..

[30] Field MG, Decatur CL, Kurtenbach S, Gezgin G, van der Velden PA, Jager MJ, Kozak KN, Harbour JW (2016). PRAME as an independent biomarker for metastasis in uveal melanoma. Clin Cancer Res.

[31] Tan P, Zou C, Yong B, Han J, Zhang L, Su Q, Yin J, Wang J, Huang G, Peng T (2012). Expression and prognostic relevance of PRAME in primary osteosarcoma. Biochem Biophys Res Commun.

[32] Szczepanski MJ, DeLeo AB, Łuczak M, Molinska-Glura M, Misiak J, Szarzynska B, Dworacki G, Zagor M, Rozwadowska N, Kurpisz M (2013). PRAME expression in head and neck cancer correlates with markers of poor prognosis and might help in selecting candidates for retinoid chemoprevention in pre-malignant lesions. Oral Oncol.

[33] Epping MT, Wang L, Edel MJ, Carlée L, Hernandez M, Bernards R (2005). The human tumor antigen PRAME is a dominant repressor of retinoic acid receptor signaling. Cell.

[34] Tanaka N, Wang Y-H, Shiseki M, Takanashi M, Motoji T (2011). Inhibition of PRAME expression causes cell cycle arrest and apoptosis in leukemic cells. Leuk Res.

[35] Zhu H, Wang J, Yin J, Lu B, Yang Q, Wan Y, Jia C (2018). Downregulation of PRAME suppresses proliferation and promotes apoptosis in hepatocellular carcinoma through the activation of P53 mediated pathway. Cell Physiol Biochem.

[36] De Carvalho DD, Mello BP, Pereira WO, Amarante-Mendes GP (2013). PRAME/EZH2-mediated regulation of TRAIL: a new target for cancer therapy. Curr Mol Med.

[37] Kewitz S, Staege MS (2013). Knock-down of PRAME increases retinoic acid signaling and cytotoxic drug sensitivity of Hodgkin lymphoma cells. PLoS ONE.

[38] Huang Q, Wei H, Wu Z, Li L, Yao L, Sun Z, Li L, Lin Z, Xu W, Han S (2016). Preferentially expressed antigen of melanoma prevents lung cancer metastasis. PLoS ONE.

[39] Sun Z, Wu Z, Zhang F, Guo Q, Li L, Li K, Chen H, Zhao J, Song D, Huang Q (2016). PRAME is critical for breast cancer growth and metastasis. Gene.

[40] Tang Z, Li C, Kang B, Gao G, Li C, Zhang Z (2017). GEPIA: a web server for cancer and normal gene expression profiling and interactive analyses. Nucleic Acids Res.

[41] Tajeddine N, Gala J-L, Louis M, Van Schoor M, Tombal B, Gailly P (2005). Tumor-associated antigen preferentially expressed antigen of melanoma (PRAME) induces caspase-independent cell death in vitro and reduces tumorigenicity in vivo. Cancer Res.

[42] Lamouille S, Xu J, Derynck R (2014). Molecular mechanisms of epithelial–mesenchymal transition. Nat Rev Mol Cell Biol.

[43] Li D, Masiero M, Banham AH, Harris AL (2014). The Notch Ligand Jagged1 as a target for anti-tumor therapy. Front Oncol.

[44] Zhan T, Rindtorff N, Boutros M (2017). Wnt signaling in cancer. Oncogene.

[45] Mani SA, Yang J, Brooks M, Schwaninger G, Zhou A, Miura N, Kutok JL, Hartwell K, Richardson AL, Weinberg RA (2007). Mesenchyme Forkhead 1 (FOXC2) plays a key role in metastasis and is associated with aggressive basal-like breast cancers. Proc Natl Acad Sci USA.

[46] Dravis C, Spike BT, Harrell JC, Johns C, Trejo CL, Southard-Smith EM, Perou CM, Wahl GM (2015). Sox10 regulates stem/progenitor and mesenchymal cell states in mammary epithelial cells. Cell Rep..

[47] Cimino-Mathews A, Subhawong AP, Elwood H, Warzecha HN, Sharma R, Park BH, Taube JM, Illei PB, Argani P (2013). Neural crest transcription factor Sox10 is preferentially expressed in triple-negative and metaplastic breast carcinomas. Hum Pathol.

[48] Reedijk M, Pinnaduwage D, Dickson BC, Mulligan AM, Zhang H, Bull SB, O’Malley FP, Egan SE, Andrulis IL (2008). JAG1 expression is associated with a basal phenotype and recurrence in lymph node-negative breast cancer. Breast Cancer Res Treat.

[49] Sánchez-Tilló E, de Barrios O, Siles L, Cuatrecasas M, Castells A, Postigo A (2011). β-catenin/TCF4 complex induces the epithelial-to-mesenchymal transition (EMT)-activator ZEB1 to regulate tumor invasiveness. Proc Natl Acad Sci USAbo.

[50] Mori H, Yao Y, Learman BS, Kurozumi K, Ishida J, Ramakrishnan SK, Overmyer KA, Xue X, Cawthorn WP, Reid MA (2016). Induction of WNT11 by hypoxia and hypoxia-inducible factor-1α regulates cell proliferation, migration and invasion. Sci Rep..

[51] Fujiwaki R, Iida K, Nakayama K, Kanasaki H, Hata K, Katabuchi H, Okamura H, Miyazaki K (2003). Clinical significance of interleukin-1 receptor antagonist in patients with cervical carcinoma. Gynecol Oncol.

[52] Iizuka N, Hazama S, Hirose K, Abe T, Tokuda N, Fukumoto T, Tangoku A, Oka M (1999). Interleukin-1 receptor antagonist mRNA expression and the progression of gastric carcinoma. Cancer Lett.

[53] Nisticò P, Bissell MJ, Radisky DC (2012). Epithelial-mesenchymal transition: general principles and pathological relevance with special emphasis on the role of matrix metalloproteinases. Cold Spring Harb Perspect Biol..

[54] Ying X, Sun Y, He P (2015). Bone morphogenetic protein-7 inhibits EMT-associated genes in breast cancer. Cell Physiol Biochem.

[55] Buijs JT, Henriquez NV, van Overveld PGM, van der Horst G, Que I, Schwaninger R, Rentsch C, Ten Dijke P, Cleton-Jansen A-M, Driouch K (2007). Bone morphogenetic protein 7 in the development and treatment of bone metastases from breast cancer. Cancer Res.

[56] Huang H, Groth J, Sossey-Alaoui K, Hawthorn L, Beall S, Geradts J (2005). Aberrant expression of novel and previously described cell membrane markers in human breast cancer cell lines and tumors. Clin Cancer Res.

[57] Huang H, Sossey-Alaoui K, Beachy SH, Geradts J (2007). The tetraspanin superfamily member NET-6 is a new tumor suppressor gene. J Cancer Res Clin Oncol.

